# Supplementary material for: Homozygosity for a Novel DOCK7 Variant Due to Segmental Uniparental Isodisomy of Chromosome 1 Associated with Early Infantile Epileptic Encephalopathy (EIEE) and Cortical Visual Impairment
Source: Int J Mol Sci. 2022 Jul 2;23(13):7382. doi: 10.3390/ijms23137382 (PMC9266905; doi:10.3390/ijms23137382)
Supplement: Supplementary file 1 [file ijms-23-07382-s001.zip › Supplementary Figure S1.pdf]

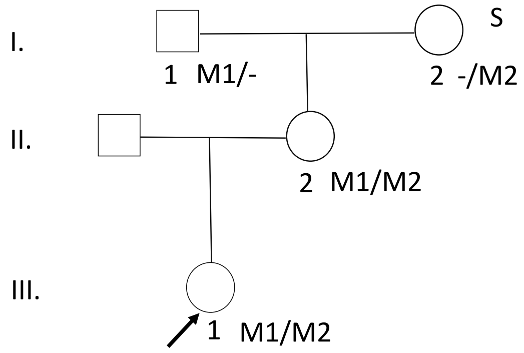

Supplementary figure S1: Pedigree structure and segregation analysis of identified *ABCA4* sequence variants in the family. Showing segregation of *ABCA4* sequence variants M1: c.157G>A (p.(Glu53Lys)) and M2: c.2267C>T (p.(Ser756Phe)). (-) indicates reference allele, whereas M1 and M2 indicate the mutant alleles. The black arrow represents the patient.
